# The dose-dependent effect of caffeine supplementation on performance, reaction time and postural stability in CrossFit – a randomized placebo-controlled crossover trial

**DOI:** 10.1080/15502783.2023.2301384

**Published:** 2024-01-16

**Authors:** Natalia Główka, Jakub Malik, Tomasz Podgórski, Rafał Stemplewski, Janusz Maciaszek, Julia Ciążyńska, Emilia E. Zawieja, Agata Chmurzynska, Paulina M. Nowaczyk, Krzysztof Durkalec-Michalski

**Affiliations:** a Department of Sports Dietetics, Poznań University of Physical Education, Poznań, Poland; b Department of Physical Activity and Health Promotion Science, Poznań University of Physical Education, Poznań, Poland; c Department of Physiology and Biochemistry, Poznań University of Physical Education, Poznań, Poland; d Department of Digital Technologies in Physical Activity, Poznań University of Physical Education, Poznań, Poland; e Department of Human Nutrition and Dietetics, Poznań University of Life Sciences, Poznań, Poland; f Sport Sciences–Biomedical Department, Faculty of Physical Education and Sport, Charles University, Prague, Czech Republic

**Keywords:** cognitive function, ergogenic support, high-intensity functional exercise, sports dietetics, stability, supplementation

## Abstract

**Background:**

Caffeine (CAF) ingestion improves performance in a broad range of exercise tasks. Nevertheless, the CAF-induced, dose-dependent effect on discipline-specific performance and cognitive functions in CrossFit/High-Intensity Functional Training (HIFT) has not been sufficiently investigated. The aim of this study was to evaluate the effect of acute supplementation of three different doses of CAF and placebo (PLA) on specific performance, reaction time (R_Time_), postural stability (P_Stab_), heart rate (HR) and perceived exertion (RPE).

**Methods:**

In a randomized double-blind placebo-controlled crossover design, acute pre-exercise supplementation with CAF (3, 6, or 9 mg/kg body mass (BM)) and PLA in 26 moderately trained CrossFit practitioners was examined. The study protocol involved five separate testing sessions using the Fight Gone Bad test (FGB) as the exercise performance evaluation and biochemical analyses, HR and RPE monitoring, as well as the assessment of R_Time_ and P_Stab_, with regard to *CYP1A2* (rs762551) and *ADORA2A* (rs5751876) single nucleotide polymorphism (SNP).

**Results:**

Supplementation of 6 mg_CAF_/kg_BM_ induced clinically noticeable improvements in FGB_Total_ results, R_Time_ and pre-exercise motor time. Nevertheless, there were no significant differences between any CAF doses and PLA in FGB_Total_, HR_max_, HR_mean_, RPE, pre/post-exercise R_Time_, P_Stab_ variables or pyruvate concentrations. Lactate concentration was higher (*p* < 0.05) before and after exercise in all CAF doses than in PLA. There was no effect of CYP1A2 or ADORA2A SNPs on performance.

**Conclusions:**

The dose-dependent effect of CAF supplementation appears to be limited to statistically nonsignificant but clinically considered changes on specific performance, R_Time_, P_Stab_, RPE or HR. However, regarding practical CAF-induced performance implications in CrossFit/HIFT, 6 mg_CAF_/kg_BM_ may be supposed as the most rational supplementation strategy.

## 1. Introduction

Caffeine (CAF) is a substance with a long history of performance-enhancing usage [1,2]. The latest umbrella review of 21 published meta-analyses on CAF supplementation and exercise performance [1] suggested that CAF ingestion improves muscle strength and endurance, anaerobic power or aerobic endurance. Although, most studies adapted standardly prescribed [2] single CAF portions (3–6 mg_CAF_/kg body mass (BM)) taken 30–60 min before exercise, the optimal doses remain still elusive, which may differ based on several factors, like exercise specificity, habitual CAF consumption, training experience, or gender [1–3]. Bounding to the adenosine receptors in the central nervous system (CNS) is the main mechanism of CAF action. Moreover, other mechanisms, like increasing myofibrillar Ca^2+^ availability or optimizing exercise metabolism and energy substrate availability, have been proposed to be responsible for ergogenic effects of CAF [2,3].

In terms of the impact of CAF in sport, it is noteworthy that its potential may be linked to the specificity of the efforts performed [2]. In this respect, particular attention is drawn to CrossFit/High-Intensity Functional Training (HIFT), which incorporates functional, multimodal movements, which are performed at high intensity and may improve general physical fitness [4,5]. It is suggested that CrossFit/HIFT training improves exercise performance, strength, aerobic and anaerobic capacity, power output, body composition, as well as resting heart rate (HR) or blood pressure [6]. There are several CrossFit/HIFT benchmark workouts (i.e. Grace, Fran, or Nancy) determining exercise performance [4,5]. One of them (Fight Gone Bad, FGB) has been validated and well-described recently, as a discipline-specific test measuring HIFT performance, by incorporating several crucial physiological traits, like stamina, strength, speed, endurance, and power [7]. Nevertheless, studies measuring the influence of dietary regimens or supplementation in CrossFit/HIFT used different exercise tests, making it difficult to compare the performance results.

Athlete’s attention, being one of the cognitive functions, is defined as the allocation of cognitive resources to internal or external stimuli. CAF can lead to the release of neurotransmitters, like dopamine and noradrenaline [8], which may promote beneficial mood changes, reduced feeling of fatigue and increased alertness. These could be also beneficial for athletes at the cognitive level [8]. Positive effects of CAF have been reported in studies examining learning speed, delayed recall, accuracy, willingness of physical effort, and reaction time (R_Time_) in response to the Stroop test and the Rapid Visual Information Processing test [9,10]. In turn, maintaining an upright stance is controlled via unconscious balance mechanisms, cortical structures and cognitive processes. CAF may influence sensorimotor functions, especially the control of standing. Muscular function and cognition aspects, like perception and attention, enhanced after CAF ingestion may positively affect postural stability (P_Stab_). On the other hand, balance may be impaired by CAF via postural disturbances caused by stimulating effects of ventilation [11].

Moreover, it is suggested that CAF ergogenicity may rely upon the genotype-modulated differences in the rate of its metabolism [3,12,13]. In this respect, it is indicated that the rs762551 single nucleotide polymorphism (SNP) of the *CYP1A2* gene can differentiate the enzyme activity into: “fast” (AA homozygotes), “slow” (AC heterozygotes) and “ultra-slow” (CC homozygotes) metabolizers [12]. In view of the fact that CAF metabolites have greater affinity to the adenosine receptors, it is suggested that faster demethylation into CAF metabolites occurs in AA homozygotes [13]. It may induce greater performance benefits by intensifying the response to CAF supplementation. In turn, it is suggested that “slow”/“ultra-slow” CAF metabolism, associated with extended periods of excessive blockage of adenosine receptors by CAF, can be detrimental to performance [3,12]. Furthermore, the other indicated gene suggested to be important in the modulation of response to CAF is *ADORA2A*, which rs5751876 SNP (three genotypes: TT and CC homozygotes, CT heterozygotes) may affect the sensitivity to CAF consumption, like the level of anxiety or emotional processing [3]. Individuals may be categorized as having a high (TT) or low (CC, CT) CAF sensitivity [14]. Nevertheless, the latest evidence do not support the hypothesis that this polymorphism in *ADORA2A* affects different performance effects [3].

Moreover, there is hardly any data on the individual dose-dependent CAF-induced changes in discipline-specific performance in CrossFit/HIFT. Therefore, the aim of our study was to evaluate the effect of three different doses of acute CAF supplementation (3, 6 and 9 mg_CAF_/kg_BM_) on specific performance, R_Time_, P_Stab_, HR and rating of perceived exertion (RPE), in a group of healthy, and moderately CrossFit/HIFT-trained athletes. Moreover, these associations may be modified by gene. For this reason, we also examined the interactions between CAF treatment dosage, FGB results and genotypes of two genes, which determine the effects of CAF on the body, namely *CYP1A2* (rs762551 A>C polymorphism) and *ADORA2A* (rs5751876 C>T polymorphism). We hypothesized that the ergogenic effects would be different and dose-dependent in all outcome measures, and that AA homozygotes would respond differently to AC heterozygotes/CC homozygotes (*CYP1A2*) and TT homozygotes differently to C-allele carriers (*ADORA2A*).

## 2. Materials and methods

### 2.1. Study design, protocol, and visits

**Figure 1.**
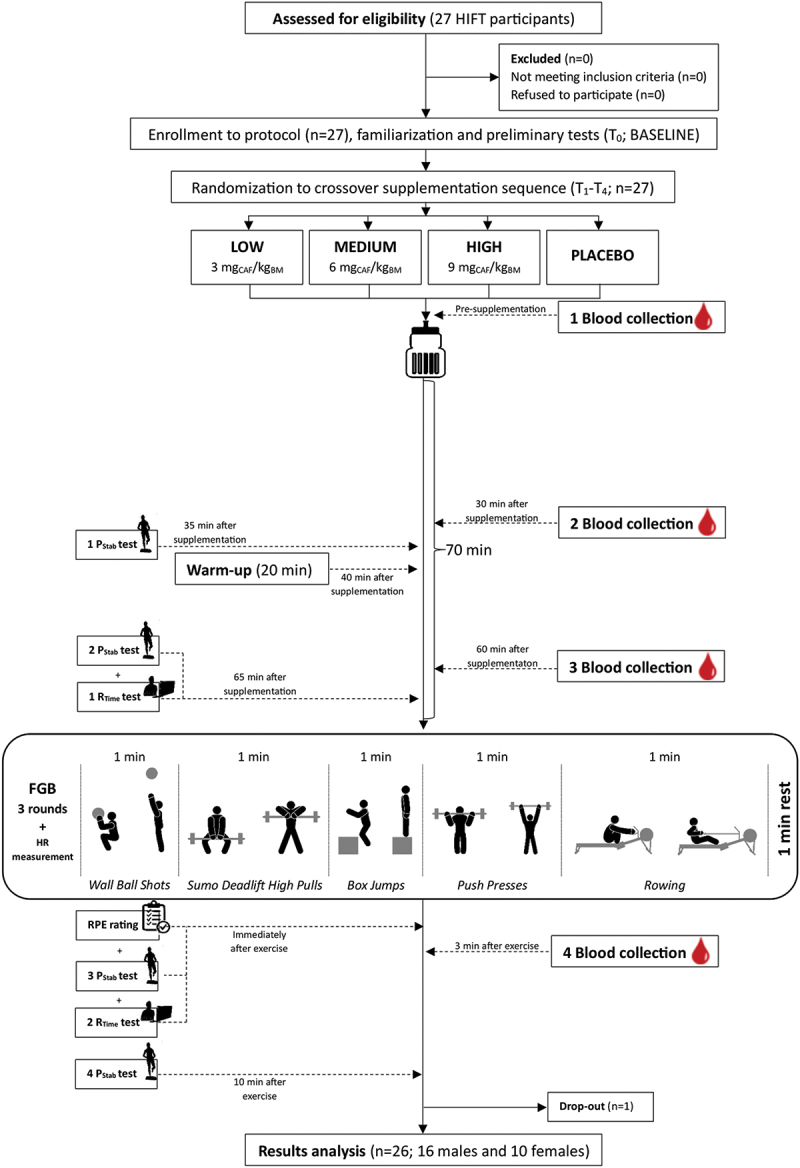
Flow chart of the study design.

FGB: Fight Gone Bad test; HIFT: High-Intensity Functional Training; HR: heart rate; P_Stab_: postural stability; RPE: the rate of perceived exertion; R_Time_: reaction time; .

The study protocol consisted of acute supplementation with three doses of CAF (3, 6, 9 mg_CAF_/kg_BM_) or PLA in a randomized double-blind placebo-controlled crossover design. The whole study protocol included five visits (T_0_–T_4_) to the laboratory (Figure 1). After the familiarization to the study protocol, enrolled volunteers were subjected to the first visit (T_0_; baseline (BASE), without supplementation treatment). After T_0_, the participants were randomly assigned (stratified randomization based on FGB test results) to the treatment order with specific codes by an impartial biostatistician. The main study protocol involving four separate visits (T_1_–T_4_) included anthropometric and body composition measurements, discipline-specific performance tests (FGB), R_Time_ and P_Stab_ examinations and blood sampling. All testing was performed at the same time of the day for the participant. A 7-day washout period was introduced between treatments, which was likely a sufficient period given the kinetics of CAF excretion from the body [15]. The participants consumed a standardized meal in compliance with our previous studies (Carbohydrates: 2 g/kg_BM_, proteins: 25 g, water: 7 mL/kg_BM_ [16,17]) 3 h before the visits. They were also instructed to avoid CAF-containing products for the 24 h preceding each test session. Moreover, participants were divided post hoc into groups according to *CYP1A2* genotype (AA homozygotes and C-allele carriers) and *ADORA2A* genotype (C-allele carriers and TT homozygotes).

The primary outcome in our study was a change in CrossFit/HIFT-specific performance, R_Time_, and P_Stab_. In turn, HR, RPE, lactate and pyruvate concentrations, and SNPs in *CYP1A2* and *ADORA2A* genes were defined as secondary outcomes.

### 2.2. Participants

Twenty-seven moderately CrossFit/HIFT-trained participants were initially enrolled in this study. Finally, 26 of them (10 females, 16 males) completed the entire study protocol and were included in the analyses (Figure 1; Table 1). All moderately trained athletes were members of the CrossFit clubs from Poznań. The criteria for qualifying for the study included good health condition, a valid and up-to-date medical certificate confirming the athlete’s ability to practice sports, at least 2 years of CrossFit/HIFT training experience, a minimum of 4 workout sessions a week, and a habitual moderate intake of CAF-containing products (participants ingested approximately 231 mg of CAF each day, which refers to more than two 240 mL cups of coffee) [18,19]. Both males and females were included because of the participation of both sexes in CrossFit/HIFT training. The study protocol was conducted in a few waves from July 2021 to December 2022 at the Department of Sports Dietetics (Poznań University of Physical Education, Poland). All athletes declared that they had not introduced any changes in their lifestyles, elements of training, nutrition or supplementation during the study protocol and that they were prepared for each study visit in the same manner, paying attention especially to the 24 h before the visit. This trial was reviewed and approved by the Bioethics Committee at Poznan University of Medical Sciences (reference number 293/17 of 11 May 2017) and was registered prospectively at ClinicalTrials.gov (NCT03822663). The study complies with the CONSORT Statement for randomized trials as shown in Figure 1. All study participants gave written informed consent. All procedures were carried out in accordance with the ethical standards of the Helsinki Declaration of 2013.

**Table 1.** Baseline characteristics of the studied group (*n* = 26).

Variable	Mean±SD
Age [years]	35.4 ± 6.5
Body mass [kg]	77.4 ± 16.6
Height [cm]	175 ± 9
Total Body Water [%, *L*]	57.2 ± 5.1, *44.1 ± 9.7*
Fat Mass [%, *kg*]	19.9 ± 5.6, *15.5 ± 6.1*
Fat-Free Mass [%, *kg*]	80.1 ± 5.7, *61.9 ± 13.6*
Energy intake [kcal, *kcal/kg*_*BM*_]	2362 ± 653, *31.0 ± 9.4*
Carbohydrates intake [g, *g/kg*_*BM*_]	281 ± 89, *3.1 ± 1.2*
Protein intake [g, *g/kg*_*BM*_]	125 ± 42, *1.6 ± 0.5*
Fat intake [g, *g/kg*_*BM*_]	84 ± 28, *1.1 ± 0.45*

SD – standard deviation; BM – body mass.

### 2.3. Procedures

#### 2.3.1. Supplementation

In the experimental procedure, each athlete was supplemented with an acute dose of CAF (3- [*LOW*], 6- [*MEDIUM*] or 9 mg_CAF_/kg_BM_ [*HIGH*]) and PLA treatment in a crossover regimen. CAF doses (pure pharmaceutical CAF, Ostrovit, Poland) and PLA (bitter aroma) were administered dissolved in 20 mL of orange juice and 80 mL of plain water, for masking the possibility of a bitter taste. On the testing days, the supplements were taken 70 min before the discipline-specific exercise test, immediately after the first blood sampling (Figure 1). The preparations were administrated to each participant in containers marked with a unique code. In accordance with the recommended blinding procedure, the preparations were made in advance by the researcher who did not directly participate in the investigations. Regarding double blinding, neither the researchers nor the participants knew whether CAF or PLA was administered. Randomization details were anonymized and revealed after the protocol cessation. The preparations were administered at a strictly specified time before the exercise tests, and consumption compliance was controlled by the investigators.

#### 2.3.2. Anthropometric and body composition measurements

Anthropometric measurements were taken at the beginning of each study visit to ensure the same conditions for the testing procedures. Prior to body composition analysis, BM and height were measured in duplicate using a calibrated scale with a stadiometer (WPT 60/150 OW, Radwag®, Radom, Poland). The total body water was assessed by bioelectric impedance with Bodystat 1500 (Bodystat Inc., Douglas, UK). Only properly hydrated participants were approved for testing. During the bioimpedance analyses, the recommended measurement conditions were strictly followed. The excellent repeatability and reliability of this method for body composition analysis were previously insightfully evaluated and are published elsewhere [7].

#### 2.3.3. Nutritional assessment

During the familiarization visit, each participant filled the special questionnaire prepared for this study regarding the amount of usual CAF intake from natural products. Daily CAF intake was calculated based on databases by Nieber et al. [19]. Moreover, the use of CAF-containing supplements and the possible side effects after CAF intake were evaluated (by the questionnaire) to verify meeting the inclusion criteria. The assessment of the diets, with a special concern to the 24 h abstention to CAF-based products, was made on the basis of the open-ended dietary assessment method, where the participants self-recorded all the foods and beverages consumed, from the period of two consecutive days before each study visit. Participants were trained in the dietary recording method by a dietitian during the familiarization visit. The quantitative analysis of nutritional value of daily food rations was carried out using the dietary software, which utilizes a database developed by the National Food and Nutrition Institute in Warsaw (Kcalmar.pro software, Lublin, Poland).

#### 2.3.4. Exercise test

##### 2.3.4.1. Discipline-specific CrossFit/HIFT performance test

FGB test, being the cross-training benchmark workout test, was chosen to assess the performance of the participants, as a discipline-specific test to measure CrossFit/HIFT performance. The FGB workout was performed according to the protocol from our previous studies, and its repeatability was assessed as high [7,18,20]. Participants followed a 20 min of self-prepared warm-up right before the commencement of the test (the warm-up was always the same for each participant). The entire test lasted for 17 min ([3 rounds (R_1–3_) × 5 min] and [2 breaks × 1 min between the rounds]). Each round consisted of five exercises: *Wall Ball Shots* (9 kg for males to a height of 3 m, 6 kg for females to a height of 2.75 m), *Sumo Deadlift High Pulls* (35 kg for males, 25 kg for females), *Box Jumps* (60 cm for males, 50 cm for females), *Push Presses* (35 kg for males, 25 kg for females), and *Rowing* (a damper setting of 7 for males and females). Participants were obligated to complete as many repetitions (or calories on rowing ergometer) as possible of each exercise during 1 min and immediately switch to the next exercise. For each valid repetition, a participant needed to complete the full range of motion required for a particular exercise. Test procedures were evaluated in real time by two independent investigators, and they were additionally visually registered. During exercise tests, the researchers and coaches provided verbal encouragement. Moreover, there was a constant monitoring of the HR using a telemetric system (Polar, Finland). From the collected data, HR_mean_ and HR_max_ were determined. At the end of the FGB, athletes were asked to rate their RPE using the Borg scale (6–20) according to published recommendations [18].

#### 2.3.5. Reaction time test

The R_Time_ test was carried out twice during each study visit (1^st^ − 65 min after supplementation, pre-exercise; 2^nd^ – immediately after exercise) using the Vienna Test System (Viennese Test System by Schuhfried GmbH, Austria; Polish distribution – COGNIFIC). The Vienna Test System is a tool commonly used and recommended in the studies of cognitive functions in athletes [21]. This system allowed to determine the participant’s R_Time_ with the accuracy of thousandths of a second. The test enabled a reliable assessment of the R_Time_ to the emerging stimulus; performing a specific reaction in the presence of various stimuli, including selection of these stimuli, while maintaining the effective R_Time_. R_Time_ test was performed using a reaction panel – one of the reaction devices of the Vienna Test System with the resting and reaction key enabling splitting the variables into reaction (R_Time_) and motor time (M_Time_) in ms. The term R_Time_ referred to the time between the presentation of the stimulus and the occurrence of the response, which meant raising the finger from the resting button. In turn, M_Time_ referred to the time between raising a finger from the rest button and pressing the R_Time_ button. Moreover, variability of R_Time_ and M_Time_ was analyzed, which refers to inconsistency in an individual’s time of responding. Participants reacted as quickly as possible to optical signals. The S1 test form the task chosen for the study from a battery of R_Time_ Vienna System tests concerned only one stimulus, lasted 4 min and included pressing the button when presented with a simple yellow light signal. The participant was instructed to use only one finger of the dominant hand. To standardize the measurements, each approach was preceded by a practice test, after which the participant performed a 4 min main test.

#### 2.3.6. Postural stability test

The P_Stab_ was assessed four times (1^st^ − 35 min after supplementation, before warm-up; 2^nd^ − 65 min after supplementation, pre-exercise; 3^rd^ – immediately after exercise; 4^th^ − 10 min after the FGB test) during each study visit on a AccuGait™ posturography platform (AMTI PJB-101 model, AMTI, Watertown, MA, USA) with Balance Trainer software and sampling frequency 100 Hz. The platform allowed to monitor changes in ground reaction forces and, on this basis, assess the position and oscillation of the center of pressure of the feet (COP). The platform was placed on a flat, hard surface. Participants were barefoot for the measurements. Each time before the start of the test, participants were instructed with the same command: “*Please stand freely on the platform – one leg, with your arms along your torso, eyes open, gaze straight ahead*” [22]. The participants stood on the dominant leg, which was previously determined by asking: ”*If you were shooting a ball at a target, which leg would you use to kick it?*.” The answer to this question indicated leg dominance in the mobilization task, as well as in stabilization tasks in 66.7% of males and 85.0% of females [23]. Each test lasted for 30 s. COP velocity (Vcop) and area 95 percentile (Area95) were analyzed as the indicators of the P_Stab_.

#### 2.3.7. Blood collection and sample analysis

Fingertip capillary blood samples were obtained at four time-points (1^st^ – pre-supplementation; 2^nd^ − 30 min after supplementation; 3^rd^ − 60 min after supplementation, pre-exercise; 4^th^ − 3 min after exercise) during each study visit. Blood samples (50 μL) were taken and immediately transferred to microtubes containing 250 μL of 0.6 M perchloric acid. Lactate (*La*) and pyruvate (*Pa*) measurements were performed according to the method used in our previous study [20].

#### 2.3.8. CYP1A2 and ADORA2A genotyping

Buccal swabs for *CYP1A2* (rs762551) and *ADORA2A* (rs5751876) genotyping were collected on the first study visit. The participants were instructed to insert the swab into the mouth and rub firmly against the inside of the cheek or underneath lower and upper lip. Rubbing lasted for 1 min. DNA was isolated from exfoliated buccal epithelial cells using a standard kit (EXTRACTME® Genomic DNA Kit EM13, Blirt SA, Poland). Genotyping was performed as described previously [24], using commercially available TaqMan®SNP genotyping assays (ThermoFisher Scientific, Waltham, MA, USA, ID C___8881221_40 and C___2446672_50) on a LightCycler 480 instrument (Roche Diagnostics, Switzerland).

### 2.4. Statistical analysis

G*Power software (version 3.1.9.6, Germany) was used to calculate minimum sample size to obtain a power greater than 0.8 with an assumed probability of alpha error of 0.05. For the purposes of this research design, minimum sample size estimation by F-test – ANOVA RM within factors was used. A *medium* effect size (ES): f (U) = 0.38 was estimated based on data from umbrella review [1] (concerning data on aerobic and muscle endurance). Using this ES, one number of groups, five measurement points, nonsphericity correction ϵ equal to one, and “as in SPSS” option analysis indicated that a sample size of 23 participants would be adequate to minimize Type I and Type II errors. For Friedman’s ANOVA, the sample size was 26, assuming the need for a 15% larger sample. The analysis was performed in Statistica 13.3 (TIBCO Software Inc. 2017) and RStudio using the DescTools package (Posit Software, PBC 2009–2023). The normality of the distribution of the variables was tested by comparing the values of skewness and kurtosis with the standard errors of these values (a value of skewness or kurtosis exceeding 1.96 standard error for a sample of less than 50 participants is considered not to meet the normality condition) [25]. Comparisons between means were made using ANOVA with repeated measures (when normality condition was met) or Friedman’s ANOVA (when normality condition was not met). Post-hoc comparisons were made using the Bonferroni test or Wilcoxon pairwise comparisons depending on whether the normality condition was met or not. Mauchly’s sphericity test was also performed to check the equality of variances of different levels. If the hypothesis of meeting the sphericity condition was rejected, the Greenhouse–Geisser correction was performed. An alpha of <0.05 was taken as a statistically significant value. The ES – partial eta square was interpreted as follows: <0.01 *no* effect; 0.01–0.06 *small* effect; 0.06–0.14 *moderate* effect; >0.14 *large* effect. In turn, Kendall W was as follows: <0.1 *no* effect; 0.1–0.3 *small* effect; 0.3–0.5 *moderate* effect; >0.5 *large* effect. In addition, a correlation analysis was conducted between the post-exercise R_Time_, M_Time_, Vcop and Area95 and RPE, FGB_Total_ and HR_max_ using Spearman’s rho correlation coefficient.

## 3. Results

### 3.1. Discipline-specific CrossFit/HIFT performance test

There were significantly more repetitions in FGB_Total_ in all treatments compared to BASE. Although, there were no significant differences between different CAF doses or PLA, *MEDIUM* CAF dose revealed clinically considerable effectiveness in improving FGB_Total_ scores. Moreover, from the practical point of view, the highest number of repetitions in R_1_ and R_2_ was observed at 6 mg_CAF_/kg_BM_. However, considering statistical significance and FGB rounds’ results separately, in R_1_, the highest number of repetitions was observed at 6 and 9 mg_CAF_/kg_BM_ (and significantly higher compared to PLA and BASE, but comparable to the effect of 3 mg_CAF_/kg_BM_). During R_2_, the number of repetitions improved in all treatments compared to BASE; and despite the same median value, 9 mg_CAF_/kg_BM_ was less effective compared to PLA (but comparable to 3 and 6 mg_CAF_/kg_BM_). During R_3_, the number of repetitions improved in all treatments compared to BASE; and PLA was significantly more effective than 9 mg_CAF_/kg_BM_, but comparable to 3 and 6 mg_CAF_/kg_BM_ (Table 2).

**Table 2.** Discipline-specific CrossFit/HIFT performance test - *Fight Gone Bad* (FGB) results.

FGB	TREATMENT					
	BASELINE Me±IQR	LOW Me±IQR	MEDIUM Me±IQR	HIGH Me±IQR	PLA Me±IQR	*X*^*2*^ [p-value]; W
Round 1						
*Wall Ball Shots* [reps]	30.0 ± 6.0	29.5 ± 7.0	30.5 ± 6.0	29.0 ± 6.0	29.0 ± 7.0	1.42 [0.84]; 0.01
*Sumo Dead Lift High Pulls* [reps]	26.5 ± 8.0	25.5 ± 9.0	26.5 ± 4.0	27.0 ± 8.0	25.0 ± 6.0	5.23 [0.26]; 0.05
*Box Jumps* [reps]	19.0 ± 4.0	18.5 ± 4.0	19.0 ± 5.0	20.0 ± 4.0	17.5 ± 6.0	3.23 [0.52]; 0.03
*Push Presses* [reps]	20.0 ± 5.0^LMHP^	22.0 ± 10.0^B^	23.5 ± 9.0^B^	25.0 ± 9.0^B^	22.0 ± 8.0^B^	24.75 [<0.01]; 0.24*
*Rowing* [cal]	15.5 ± 6.0^H^	16.0 ± 6.0	15.5 ± 5.0	16.5 ± 6.0^B^	15.0 ± 6.0	5.07 [0.28]; 0.05
Total [reps]	109.0 ± 24.0^MH,23^	110.5 ± 22.0^23^	114.5 ± 17.0^BP,23^	114.5 ± 23.0^BP,23^	110.0 ± 22.0^MH,23^	12.83 [0.01]; 0.12*
Round 2						
*Wall Ball Shots* [reps]	22.5 ± 7.0^LMHP^	25.5 ± 9.0^B^	25.0 ± 9.0^B^	26.0 ± 9.0^B^	25.5 ± 9.0^B^	29.52 [<0.01]; 0.28*
*Sumo Dead Lift High Pulls* [reps]	17.0 ± 6.0^LMHP^	20.0 ± 6.0^B^	21.0 ± 7.0^B^	21.5 ± 8.0^B^	20.0 ± 6.0^B^	28.49 [<0.01]; 0.27*
*Box Jumps* [reps]	16.0 ± 5.0	17.0 ± 5.0	17.0 ± 5.0^H^	17.0 ± 4.0^M^	18.0 ± 5.0	29.14 [<0.01]; 0.28*
*Push Presses* [reps]	16.0 ± 6.0^LMHP^	17.5 ± 10.0^B^	18.5 ± 8.0^B^	17.5 ± 8.0^BP^	20.5 ± 8.0^BH^	36.02 [<0.01]; 0.35*
*Rowing* [cal]	12.0 ± 5.0^LMHP^	13.5 ± 7.0^B^	13.0 ± 4.0^B^	13.0 ± 6.0^B^	14.0 ± 6.0^B^	20.18 [<0.01]; 0.19*
Total [reps]	81.0 ± 20.0^LMHP,13^	94.0 ± 19.0^B,13^	95.0 ± 15.0^B,13^	93.5 ± 24.0^BP,13^	93.5 ± 20.0^BH,13^	47.07 [<0.01]; 0.45*
Round 3						
*Wall Ball Shots* [reps]	20.0 ± 8.0^LMHP^	21.5 ± 8.0^B^	22.0 ± 7.0^B^	20.0 ± 9.0^BP^	22.0 ± 7.0^BH^	12.17 [0.02]; 0.12*
*Sumo Dead Lift High Pulls* [reps]	15.5 ± 5.0^LMHP^	18.0 ± 6.0^B^	19.0 ± 6.0^B^	18.5 ± 6.0^B^	19.0 ± 5.0^B^	26.63 [<0.01]; 0.26*
*Box Jumps* [reps]	14.5 ± 5.0^MP^	15.5 ± 5.0	15.5 ± 5.0^B^	15.0 ± 6.0^P^	16.0 ± 6.0^BH^	8.79 [0.07]; 0.08
*Push Presses* [reps]	15.0 ± 7.0^LMP^	16.5 ± 9.0^B^	17.0 ± 6.0^B^	17.0 ± 8.0^P^	18.0 ± 10.0^BH^	15.02 [<0.01]; 0.14*
*Rowing* [cal]	12.5 ± 7.0^LHP^	15.0 ± 5.0^B^	14.0 ± 6.0	15.0 ± 6.0^B^	15.0 ± 6.0^B^	11.79 [0.02]; 0.11*
Total [reps]	78.0 ± 16.0^LMHP,12^	84.5 ± 23.0^B,12^	86.0 ± 18.0^B,12^	83.0 ± 19.0^BP,12^	86.5 ± 24.0^BH,12^	26.36 [<0.01]; 0.25*
Rounds 1–3 *X*^*2*^ [p-value]; W	36.83 [<0.01]; 0.71*	42.57 [<0.01]; 0.82*	47.59 [<0.01]; 0.91*	49.65 [<0.01]; 0.95*	30.06 [<0.01]; 0.58*	
RPE_Total_	18.5 ± 2.0	19.0 ± 2.0	19.0 ± 2.0	19.0 ± 2.0	19.0 ± 2.0	7.32 [0.12]; 0.07
	BASELINE Mean±SD	LOW Mean±SD	MEDIUM Mean±SD	HIGH Mean±SD	PLA Mean±SD	F [p-value]; η^2^
FGB_Total_ [reps]	271 ± 45^LMHP^	290 ± 55^B^	294 ± 57^B^	293 ± 67^B^	289 ± 66^B^	19.74 [<0.01]; 0.44*
HR_mean_ during FGB [bmp]	159 ± 8^LH^	163 ± 9^B^	161 ± 10	163 ± 9^B^	161 ± 8	3.24 [0.03]; 0.11*
HR_max_ during FGB [bmp]	179 ± 10	179 ± 8	178 ± 10	177 ± 8	179 ± 11	0.59 [0.58]; 0.02

BASELINE, B – familiarization; LOW, L − 3 mg_CAF_/kg_BM_; MEDIUM, M − 6 mg_CAF_/kg_BM_; HIGH, H − 9 mg_CAF_/kg_BM_; PLA, P – placebo; Me – median; IQR – interquartile range; HR – heart rate; SD – standard deviation; *X*^*2*^ - chi-square; RPE – the rate of perceived exertion; FGB – Fight Gone Bad test; HR – heart rate. W – Kendall’s coefficient of concordance; F – f distribution; η^2^ – partial eta squared; ^BLMHP^ – different letter inscriptions refer to statistical differences between treatments (CAF doses, PLA, and baseline); ^123^ – different number inscriptions refer to statistical differences between rounds; * – statistically significant (*p* < 0.05).

Considering particular exercises, the number of *Wall Ball Shots* and *Sumo Deadlift High Pulls* was significantly higher in all treatments compared to BASE in R_2_ and R_3_. The number of *Box Jumps* was higher (*p* = 0.03) in 6 mg_CAF_/kg_BM_ than in 9 mg_CAF_/kg_BM_ in R_2_; in turn in R_3_ in 6 mg_CAF_/kg_BM_ and PLA than in BASE; and in PLA compared to 9 mg_CAF_/kg_BM_. The number of *Push Presses* in R_1_ and R_2_ was significantly higher in all conditions than in BASE, and in R_3_ in 3, 6 mg_CAF_/kg_BM_ and PLA than in BASE, and in PLA compared to 9 mg_CAF_/kg_BM_. The number of kcal in *Rowing* in R_1_ was significantly higher in 9 mg_CAF_/kg_BM_ than in BASE, in R_2_ in all treatments than in BASE, in R_3_ in 3, 9 mg_CAF_/kg_BM_ and PLA compared to BASE (Table 2).

Regarding the HR and the RPE, there were no significant differences between HR_mean_, HR_max_ or RPE and CAF/PLA during the whole study protocol. However, in comparison to BASE only HR_mean_ was higher (*p* < 0.05) after 3 and 9 mg/kg_BM_ of CAF (Table 2).

**Figure 2.**
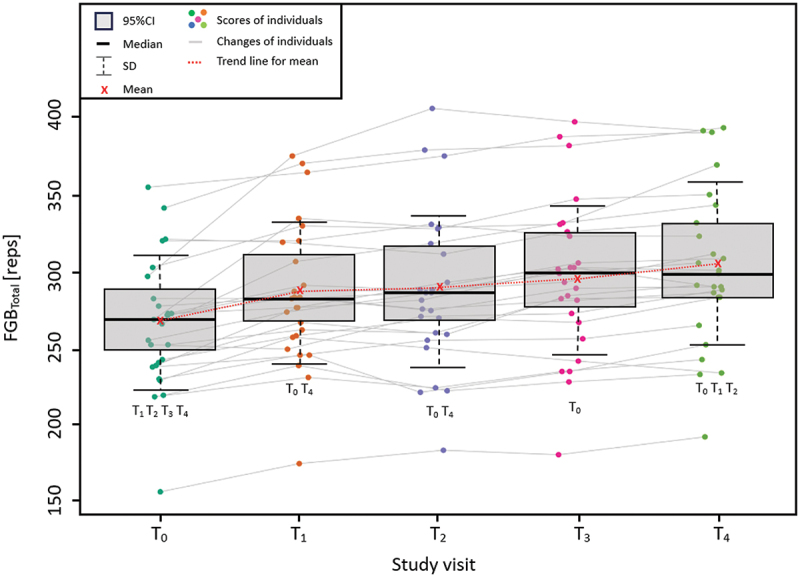
Practice effect analysis for FGB_Total_ results.

Performed practice effect analysis for FGB_Total_ revealed significant effect [F(1.83; 48.56) = 33.25, *p* < 0.05, η = 0.57] of the practice (the sequence of visits) on the number of repetitions (Figure 2).

FGB – Fight Gone Bad test; T_0_–T_4_ : order of the study visits in the laboratory.

Furthermore, in all CAF or PLA treatments, *La* and *Pa* concentrations increased significantly from the 1^st^ to the 4^th^ time-point. Pre-exercise *La* concentration was the highest in 9 mg_CAF_/kg_BM_; in turn its concentration was significantly lower in PLA compared to CAF treatments and BASE. Post-exercise *La* concentration was also the highest in 9 mg_CAF_/kg_BM_. In all CAF treatments, post-exercise *La* concentration was significantly higher compared to PLA and BASE. Between CAF treatments, significantly higher post-exercise concentration was observed in 9 compared to 3 mg_CAF_/kg_BM_ (Table 3).

**Table 3.** Lactate and pyruvate concentrations’ results.

	TREATMENT					
	BASELINE Me±IQR	LOW Me±IQR	MEDIUM Me±IQR	HIGH Me±IQR	PLA Me±IQR	*X*^*2*^ [p-value]; W
Lactate concentration [mmol/L]						
Pre-supplementation	1.15 ± 0.73	1.59 ± 0.75	1.55 ± 1.0	1.55 ± 0.82	1.51 ± 0.82	2.06 [0.72]; 0.02
30 min after supplementation	1.18 ± 0.57	1.33 ± 0.78	1.52 ± 0.66	1.39 ± 0.52	1.42 ± 0.69	6.44 [0.17]; 0.06
60 min after supplementation_pre-exercise_	2.63 ± 1.95^P^	2.89 ± 1.39^P^	2.87 ± 1.59^P^	3.04 ± 2.56^P^	1.96 ± 1.10^BLMH^	12.34 [0.02]; 0.12*
3 min after exercise	14.42 ± 3.01^LMHP^	17.01 ± 2.63^BHP^	17.40 ± 4.45^BP^	17.69 ± 3.73^BLP^	15.34 ± 3.20^BLMH^	38.06 [<0.01]; 0.47*
Pyruvate concentration [mmol/L]						
Pre-supplementation	0.16 ± 0.10	0.18 ± 0.14	0.20 ± 0.15	0.20 ± 0.13	0.22 ± 0.14	4.18 [0.38]; 0.04
30 min after supplementation	0.19 ± 0.14	0.18 ± 0.12	0.21 ± 0.12	0.19 ± 0.12	0.21 ± 0.14	4.33 [0.36]; 0.04
60 min after supplementation_pre-exercise_	0.26 ± 0.16	0.25 ± 0.07	0.29 ± 0.14	0.28 ± 0.16	0.22 ± 0.19	4.64 [0.33]; 0.04
3 min after exercise	0.49 ± 0.40	0.45 ± 0.30	0.61 ± 0.27	0.49 ± 0.38	0.57 ± 0.40	6.85 [0.14]; 0.07

BASELINE, B – familiarization; LOW, L − 3 mg_CAF_/kg_BM_; MEDIUM, M − 6 mg_CAF_/kg_BM_; HIGH, H − 9 mg_CAF_/kg_BM_; PLA, P – placebo; Me – median; IQR – interquartile range; *X*^*2*^ – chi-square; W – Kendall’s coefficient of concordance; ^BLMHP^ – different letter inscriptions refer to statistical differences between treatments (CAF doses, PLA, and baseline); * – statistically significant (*p* < 0.05).

### 3.2. CYP1A2/ADORA2A polymorphisms and FGB performance test

With regard to *CYP1A2* polymorphism, there were six fast metabolizers (AA homozygotes) and twenty “slow metabolizers” (C-allele carriers: 3 CC, 17 AC). With regard to *ADORA2A* polymorphism, there were four participants having high sensitivity (TT homozygotes) and twenty-two having low sensitivity to CAF (C-allele carriers: 10 CC, 12 CT). There were no significant interactions with *CYP1A2* or *ADORA2A* genotype or CAF dose with FGB_Total_ result (Table 4).

**Table 4.** Measurements of FGB_Total_ in participants with different *CYP1A2* (rs762551) and *ADORA2A* genotypes (rs5751876).

	TREATMENT					
	BASELINE Mean±SD	LOW Mean±SD	MEDIUM Mean±SD	HIGH Mean±SD	PLA Mean±SD	F [p-value]; η^2^
FGB_Total_ by *CYP1A2* genotype						
Fast (*n* = 6, AA) [reps]	250 ± 23	261 ± 29	271 ± 33	277 ± 30	271 ± 28	1.96 [0.14]; 0.08
Slow (*n* = 20, 3 CC, 17 AC) [reps]	274 ± 47	306 ± 57	306 ± 50	300 ± 55	305 ± 58	
FGB_Total_ by *ADORA2A* genotype						
High (*n* = 4, TT) [reps]	260 ± 82	268 ± 68	284 ± 68	271 ± 71	276 ± 69	1.30 [0.28]; 0.05
Low (*n* = 22,10 CC, 12 CT) [reps]	270 ± 36	300 ± 52	301 ± 45	299 ± 47	301 ± 52	

BASELINE, B – familiarization; LOW − 3 mg_CAF_/kg_BM_; MEDIUM − 6 mg_CAF_/kg_BM_; HIGH − 9 mg_CAF_/kg_BM_; PLA, P – placebo; FGB – Fight Gone Bad test; SD – standard deviation.

F – f distribution; η^2^ – partial eta squared; *p* < 0.05.

### 3.3. Reaction time and postural stability tests

The shortest pre- and post-exercise R_Time_ were observed in 6 mg_CAF_/kg_BM_, but the only significant difference was observed in pre-exercise R_Time_ in BASE compared to 9 mg_CAF_/kg_BM_ (significantly slower). The significant change from Pre_FGB_ to Post_FGB_ R_Time_ was observed only in 9 mg_CAF_/kg_BM_ (significantly slower post exercise). The lowest pre-exercise variability R_Time_ was observed in BASE and 3 mg_CAF_/kg_BM_; in turn post-exercise in 6 mg_CAF_/kg_BM_, but there were no significant differences between treatments. Beneficial lack of deterioration was observed only in 6 mg_CAF_/kg_BM_, whereas the significant change from Pre_FGB_ to Post_FGB_ variability R_Time_ was observed in 3, 9 mg_CAF_/kg_BM_, PLA and BASE (Table 5).

**Table 5.** Reaction time and postural stability results.

	TREATMENT					
	BASELINE Me±IQR	LOW Me±IQR	MEDIUM Me±IQR	HIGH Me±IQR	PLA Me±IQR	*X*^*2*^ [p-value]; W
REACTION TIME (R_Time_)						
Pre_FGB_ [ms]	254.0 ± 55.0^H^	249.5 ± 35.0	243.5 ± 47.0	250.5 ± 35.0^B^	250.0 ± 42.0	10.08 [0.04]; 0.10*
Post_FGB_ [ms]	251.0 ± 53.0	249.0 ± 67.0	246.5 ± 59.0	251.0 ± 50.0	252.0 ± 73.0	0.56 [0.97]; 0.01
Pre_FGB_-Post_FGB_ d_ME_ [95%CI]; p-value	3.0 [−9.0;15.0]; 0.51	1.0 [−14.0;5.0]; 0.48	−4.0 [−17.0;1.0]; 0.05	−11.5 [−24.0;-5.0]; 0.01*	1.5 [−17.0;9.0]; 0.45	
Variability Pre_FGB_ [ms]	25.0 ± 9.0	25.0 ± 12.0	27.5 ± 9.0	27.5 ± 17.0	27.0 ± 9.0	2.54 [0.64]; 0.02
Variability Post_FGB_ [ms]	31.0 ± 17.0	34.0 ± 15.0	30.0 ± 13.0	31.5 ± 13.0	30.5 ± 12.0	2.06 [0.73]; 0.02
Variability Pre_FGB_-Post_FGB_ d_ME_ [95%CI]; p-value	−5.0 [−11.0;-3.0]; <0.01*	−6.0 [−13.0;-2.0]; <0.01*	−2.5 [−8.0;1.0]; 0.07	−4.0 [−8.0;0.0]; <0.01*	−3.5 [−6.0;0.0]; 0.04*	
MOTOR TIME (M_Time_)						
Pre_FGB_ [ms]	111.0 ± 34.0^LMH^	108.0 ± 37.0^B^	110.0 ± 42.0^B^	102.0 ± 42.0^B^	109.0 ± 45.0	13.71 [<0.01]; 0.13*
Post_FGB_ [ms]	101.0 ± 59.0	104.5 ± 46.0	110.0 ± 51.0	107.5 ± 5.0	108.0 ± 59.0	3.18 [0.53]; 0.03
Pre_FGB_-Post_FGB_ d_ME_ [95%CI]; p-value	3.0 [0.0;17.0]; 0.16	−9.0 [−21.0;3.0]; <0.01*	−7.0 [−19.0;0.0]; 0.02*	−9.0 [−18.0;1.0]; 0.01*	−6.0 [−17.0;1.0]; 0.04*	
Variability Pre_FGB_ [ms]	15.5 ± 7.0^LMHP^	12.0 ± 6.0^B^	11.5 ± 5.0^B^	12.0 ± 5.0^B^	13.0 ± 5.0^B^	16.17 [<0.01]; 0.16*
Variability Post_FGB_ [ms]	15.5 ± 10.0	15.0 ± 7.0	14.0 ± 6.0	14.5 ± 6.0	16.0 ± 8.0	4.57 [0.33]; 0.04
Variability Pre_FGB_-Post_FGB_ d_ME_ [95%CI]; p-value	0.0 [−6.0;2.0]; 0.59	−3.0 [−5.0;-2.0]; <0.01*	−1.5 [−7.0;0.0]; 0.01*	−2.5 [−5.0;1.0]; 0.01*	−2.0 [−5.0;-1.0]; <0.01*	
COP VELOCITY (Vcop)						
1 (35 min after supplementation) [cm/s]	3.95 ± 1.09^P,34^	3.68 ± 1.02^MP,34^	3.93 ± 1.27^LP,34^	3.99 ± 1.01^P,34^	3.47 ± 1.04^BLMH,34^	20.22 [<0.01]; 0.19*
2 (65 min after supplementation, pre-exercise) [cm/s]	3.63 ± 1.31^34^	3.65 ± 1.20^34^	3.71 ± 1.08^34^	3.89 ± 0.82^34^	3.64 ± 0.96^34^	6.54 [0.16]; 0.06
3 (immediately after exercise) [cm/s]	5.63 ± 1.85^124^	5.07 ± 1.73^124^	5.45 ± 1.82^124^	5.20 ± 1.73^124^	4.93 ± 1.34^124^	5.26 [0.26]; 0.05
4 (10 min after exercise) [cm/s]	4.5 ± 1.36^123^	4.40 ± 1.24^123^	4.26 ± 1.64^123^	4.55 ± 1.67^123^	4.14 ± 0.94^123^	7.72 [0.10]; 0.07
Total 1–4 *X*^*2*^ [p-value]; W	44.77 [<0.01]; 0.57*	46.06 [<0.01]; 0.59*	42.97 [<0.01]; 0.55*	44.45 [<0.01]; 0.57*	5.17 [<0.01]; 0.64*	
COP AREA 95% (Area95)						
1 (35 min after supplementation) [cm^2^]	8.90 ± 3.88^34^	7.97 ± 3.78^3^	8.45 ± 2.79^3^	8.51 ± 5.27^3^	8.09 ± 5.58^3^	1.55 [0.82]; 0.01
2 (65 min after supplementation, pre-exercise) [cm^2^]	7.06 ± 5.34^34^	8.66 ± 5.53^3^	8.40 ± 3.86^3^	7.92 ± 5.24^3^	7.71 ± 2.58^3^	2.98 [0.56]; 0.03
3 (immediately after exercise) [cm^2^]	14.75 ± 8.20^124^	14.13 ± 9.28^124^	13.87 ± 6.48^124^	13.99 ± 5.04^124^	10.79 ± 7.95^124^	3.82 [0.43]; 0.04
4 (10 min after exercise) [cm^2^]	9.60 ± 6.05^123^	8.99 ± 5.20^3^	8.65 ± 4.02^3^	8.32 ± 5.05^3^	8.87 ± 4.14^3^	3.29 [0.51]; 0.03
Total 1–4 *X*^*2*^ [p-value]; W	39.65 [<0.01]; 0.51*	26.41 [<0.01]; 0.34*	33.72 [<0.01]; 0.43*	29.68 [<0.01]; 0.38*	21.09 [<0.01]; 0.27*	

BASELINE, B – familiarization; LOW, L − 3 mg_CAF_/kg_BM_; MEDIUM, M − 6 mg_CAF_/kg_BM_; HIGH, H − 9 mg_CAF_/kg_BM_; PLA, P – placebo; Me – median; IQR – interquartile range; SD – standard deviation; X2 – chi-square; W – Kendall’s coefficient of concordance; 95%Cl − 95% confidence interval; dME – median difference; p – p-value; FGB – Fight Gone Bad exercise test; PreFGB − 65 min after supplementation, pre-exercise; PostFGB – immediately after exercise; 1 – 35 min after supplementation, 2 – 65 min after supplementation, pre-exercise, 3 – immediately after exercise, 4 – 10 min after exercise; BLMHP – different letter inscriptions refer to statistical differences between CAF doses; 123 – different number inscriptions refer to statistical differences between different time-points of Vcop or Area95; * – statistically significant (p-value <0.05)

The shortest pre-exercise M_Time_ was observed in 6 mg_CAF_/kg_BM_ and post-exercise in 3 mg_CAF_/kg_BM_, but there were no significant differences between treatments. The lowest pre- and post-exercise variability M_Time_ was observed in 6 mg_CAF_/kg_BM_; there was a significantly higher pre-exercise variability M_Time_ in BASE compared to all other treatments. The significant change from Pre_FGB_ to Post_FGB_ M_Time_ and variability M_Time_ was observed in all CAF/PLA treatments, but not in BASE (Table 5).

There were significant differences in Vcop and Area95 between time-points in every condition. The worst results (the highest Area95 and Vcop) were recorded immediately post-exercise (time-point 3: Vcop, Area95). There were no statistically significant differences at this time-point between CAF doses, PLA and BASE. Vcop_1_ (35 min after supplementation) was statistically higher in all CAF treatments and BASE compared to PLA; moreover 3 mg_CAF_/kg_BM_ was characterized by higher velocity of COP than 6 mg_CAF_/kg_BM_ (Table 5).

### 3.4. Correlations

There were small positive correlations between HR_max_ and: a) Vcop_3_, b) AREA95_3_, c) VMT_POST_, d) AREA 95_3–4_, which implies a worsening of the results as HR_max_ increased. Furthermore, small/medium negative correlations were observed between: RPE and AREA 95_3_, as well as FGB_Total_ and: a) R_Time-POST_, b) VR_Time-POST_, c) M_Time-POST_, d) VM_Time-POST_, which means that as the FGB_Total_ and RPE increased, the indicated correlated values also improved. It is suggested that if the variabilities of postural stability (Vcop and AREA95) are higher, the total postural stability is worse; and if the variabilities of R_Time_ and M_Time_ are higher, the R_Time_ is slower. This specific sequence of events (tests number 3 and 4 occurring immediately after FGB) allows to speculate the observed correlations as a cause-and-effect phenomenon (Table 6).

**Table 6.** Correlations between selected results.

	RPE rho [p-value]	FGB_Total_ rho [p-value]	HR_max_ rho [p-value]
Vcop_3_	−0.08 [0.93]	0.020 [0.84]	0.225 [0.02]*
Vcop_4_	−0.075 [0.45]	0.044 [0.65]	0.124 [0.21]
Area95_3_	−0.208 [0.03]*	−0.060 [0.55]	0.216 [0.03]*
Area95_4_	−0.102 [0.30]	−0.116 [0.24]	−0.06 [0.95]
R_Time-_Post_FGB_	0.06 [0.95]	−0.208 [0.03]*	−0.106 [0.29]
VR_Time-_Post_FGB_	0.150 [0.13]	−0.310 [0.01]*	0.030 [0.76]
M_Time-_Post_FGB_	0.059 [0.55]	−0.228 [0.02]*	−0.072 [0.47]
VM_Time-_Post_FGB_	−0.163 [0.10]	−0.329 [<0.01]*	0.227 [0.02]*
Area95_3–4_	−0.117 [0.24]	0.086 [0.39]	0.291 [0.03]*
Vcop_3–4_	0.028 [0.78]	−0.135 [0.17]	0.192 [0.05]
RPE	-	−0.011 [0.90]	−0.093 [0.29]
HR_max_	−0.093 [0.29]	−0.014 [0.87]	-

rho – Spearman correlation coefficient; RPE – the rate of perceived exertion; FGB – Fight Gone Bad test; HR – heart rate; Vcop – COP VELOCITY; Area95 – COP AREA 95%; R_Time_ – reaction time; M_Time_ – motor time; VR_Time_ – variability of reaction time; VM_Time_ – variability of motor time;   3 – immediately after exercise, 4 – 10 min after exercise; Pre_FGB_ – 65 min after supplementation, pre-exercise; Post_FGB_ – immediately after exercise; * – statistically significant (*p* < 0.05).

## 4. Discussion

The aim of this study was to evaluate the effects of three different doses of acute CAF supplementation (3, 6 and 9 mg_CAF_/kg_BM_) in CrossFit/HIFT-trained participants. The primary outcome was the effect on specific performance, R_Time_ and P_Stab_. The secondary outcomes were HR, RPE, lactate and pyruvate concentrations, and SNPs in *CYP1A2* and *ADORA2A* genes. It was hypothesized that different doses of CAF would affect the outcomes differently and performance effects would be dependent on SNPs in *CYP1A2* and *ADORA2A* genes. The hypothesis was not supported, as no significant differences in CrossFit/HIFT performance, R_Time_ or P_Stab_ between CAF doses and its dependence on SNPs were observed. Nevertheless, from the clinical and athletic point of view the *MEDIUM* dose of CAF (6 mg_CAF_/kg_BM_) showed the highest effectiveness in improving the total number of repetitions in the whole FGB, in round 1 and 2, as well as in pre-exercise R_Time_ and M_Time_. Moreover, a significant practice effect was observed between study visits. To the best of the authors’ knowledge, this is the first study to determine the effects of three different doses of CAF on specific performance validated FGB test, R_Time_ and P_Stab_ in CrossFit/HIFT practitioners.

Currently, there are only four other [26–29] studies assessing the effects of CAF supplementation in CrossFit/HIFT-trained participants. Nevertheless, none of them used different doses of CAF and incorporated validated discipline-specific FGB exercise test to assess performance. As mentioned above, our study showed that although 6 mg_CAF_/kg_BM_ was clinically the most effective – no significant differences between different CAF doses were observed. On the other hand, no favorable effect of higher dose (9 mg_CAF_/kg_BM_) than recommended was showed. It is important to note that the whole FGB test lasted for 17 min (15 min of active work with 2 min of rest between rounds) and the *MEDIUM* CAF dose was the most effective during the first two rounds, which indicates that the highest usually recommended CAF dose in sport may be the most effective during early stages of longer CrossFit/HIFT performance or be more effective in shorter exercise tasks. Similarly to our results, Fogaca et al. [26] found no effect of 6 mg/kg_BM_ of CAF compared to PLA on CrossFit performance measured by the test based on 10 min of as many repetitions/rounds as possible (AMRAP) of double-unders and power snatches. Moreover, there was no effect on muscle strength (handgrip strength) and power (bench throw, jump squat and countermovement jump) in CrossFit participants. Furthermore, in study by Stein et al. [27] acute supplementation of 5 mg_CAF_/kg_BM_ in comparison to PLA ingested before Cindy benchmark workout did not improve performance. In another study assessing CrossFit performance by Cindy workout, Ziyaiyan et al. [28] examined the effect of 6 mg_CAF_/kg_BM_ in comparison to NaHCO_3_ ingestion separately and in combination. The results indicated that all conditions improved exercise performance compared to PLA, but did not reveal a significant effect of supplementation or any statistically significant differences between supplementations. However, this benchmark workout provides a unique high-volume muscular endurance challenge as it lasts 20 min and requires from the participants to complete AMRAP of 3 exercises (5 pull-ups, 10 push-ups, 15 air squats). The fourth study, by Caetano et al. [29] assessed the effect of 6 mg/kg_BM_ of CAF on one Repetition Maximum (RM) back squat test and local muscular endurance measured based on repetitions using a percentage derived from the 1RM test results. Authors found that local muscular endurance score and the average number of repetitions were higher for the CAF group compared to PLA.

Studies on the effect of CAF on exercise performance are abundant. Nevertheless, even though there are a lot of meta-analyses examining the same outcome, they may have produced conflicting findings. Recently, Grgic et al. [1] published an umbrella review on the ergogenicity of CAF, which helped to overcome potential limitations of previous meta-analyses. ESs of positive effects of CAF from analyzed meta-analyses ranged from *very small* to *moderate* (d Cohen) [1,30]. The results of aforementioned umbrella review were divided concerning the type of exercise. With regard to aerobic endurance, the majority of studies reported *small*–*moderate* ergogenic effects of CAF (ES range: 0.22–0.61). Analyses examining the effects of CAF on different measures of muscle strength observed *very small*–*small* ergogenic effect (ES range: 0.16–0.20). In turn, the effects of CAF on muscular endurance were reported as *small* (ES range: 0.28–0.38). Other results of ergogenic effects of CAF on mean and peak power were defined as *very small*–*small* (ES range: 0.18–0.27), on vertical jump height as *very small* (ES: 0.17), on speed running, cycling, or rowing as *small* (ES: 0.41), and on various forms of short-term high-intensity exercise (time to exhaustion, mean and peak power output, time trial) as *very small* (ES: 0.16) [1]. In summary, CAF is ergogenic for different components of exercise performance (aerobic and muscle endurance, muscle strength, power, jumping performance and exercise speed). Interestingly, it can be assumed that CAF not only provides a performance-enhancing effect on aerobic exercise performance, but also on anaerobic exercise tasks [1,3].

The importance of *CYP1A2* and *ADORA2A* SNPs on performance still remains unclear, therefore genotypic limitations that prevent CAF from exerting its ergogenic effects are constantly discussed [31]. Moreover, it is speculated that possible positive effects are higher in males compared to females and there may be inter-individual variation in CAF ergogenicity [14]. Additionally, it is important to note that because of the differences in the proportions of the individual SNPs in the world population, it is difficult to capture their actual impact. For instance, following the Hardy-Weinberg principle, the frequency of *CYP1A2* SNP in the population is approximately 45% for AAs, 45% for ACs and 10% for CCs [3].

Initial data from the meta-analysis by Barreto et al. [30] suggests that regarding rs762551 *CYP1A2* polymorphism, AA homozygotes may benefit most from CAF supplementation, whether for CC homozygotes CAF is ergolytic. Indeed, studies by Guest et al. [12], Womack et al. [32] or Wong et al. [33] support greater ergogenic benefits of CAF supplementation in fast metabolizers than in C-allele carriers. Nevertheless, the majority of studies do not support this hypothesis [24,34–37]. Regarding rs5751876 *ADORA2A* polymorphism, only one pilot study [38] showed that genotypes may have an influence on performance and C-allele carriers can be identified as non-responders, whereas in TT homozygotes intensive exercise improved. However, other studies [34,36,39] did not replicate these results, showing no beneficial effects of CAF were influenced by *ADORA2A* genotypes [3]. Similarly to other studies, our results showed no effect of *CYP1A2* or *ADORA2A* SNPs on performance, even with the implementation of different CAF doses.

The current study showed that the highest post-exercise *La* concentration was observed in the *HIGH* CAF dose (9 mg_CAF_/kg_BM_). Elevation in blood *La* often, but not always, accompanies CAF supplementation. At rest, CAF has no effect on *La*, meaning that to achieve the response, some mechanical stress is mandatory [40]. Although some studies have observed a significant increase in *La* [41,42], others have found no effect of CAF on *La* during submaximal exercise [43,44]. Even though the possible increase can be explained by a concomitant CAF-induced increase in performance [41,43], the mechanism underlying this increase is not so obvious [40]. CAF may increase *La* through adrenaline mediation or stimulation of CNS dampening pain perception, which extends the duration of exercise resulting in greater *La* accumulation [45]. Graham et al. [42] did not attribute the CAF-induced increase in *La* during submaximal exercise to an increase in production or release by the active muscles. Nevertheless, it may have been caused by the fact that CAF may impair *La* clearance; or insufficient sensitivity to detect changes in muscle *La* production and release resulting from CAF; or by the fact that *La* is being produced from inactive muscles. The first possibility is the most plausible, because, although it is hard to identify the precise mechanism, adenosine signaling has been shown to stimulate gluconeogenesis, which accounts for 20–30% of *La* clearance during exercise [40]. On the contrary to Graham et al. [42], Glaister et al. [40] found no effect of CAF on the rate of *La* clearance in recovery, suggesting that an increase in *La* efflux from the working muscles caused the elevation in blood *La*, supporting the idea of a CAF-stimulated increase in glycolysis rate. Furthermore, it is known that the conversion of *La* to *Pa* occurs substantially in working muscles and that on net bases *La* and *Pa* are released at similar rates. Moreover, arterial *La* is a major source of *Pa* released from muscles, but the majority of venous *Pa* is cleared before entry into arterial circulation [46]. Indeed, studies support the *Pa* increase during exercise [20,47]. Similar to the data from the literature, our results showed the increase in *Pa* during the study protocol, nevertheless there was no effect of different CAF doses on its concentration.

There were no differences in RPE between CAF doses and PLA. Although CAF did not affect RPE significantly, better performance after *MEDIUM* dose of CAF supplementation may suggest a central effect resulting in greater overall exercise intensity at the same RPE. It is speculated that multi-joint exercises may increase RPE [48]. The only existing meta-analysis on CAF and RPE [49] suggested that CAF RPE was lower than PLA RPE during constant load exercise, but no corresponding difference between CAF and PLA RPE at the conclusion of exhausting exercise was found. The authors explained that the reason may be due to a dampening of the perceptual response during exercise. The results of the current study incorporated the standard 6–20 Borg scale. Unfortunately, three other CrossFit CAF studies used a different, 10-point scale. Stein et al. [27] utilized a 10-point Likert scale that may have not been sensitive enough to capture changes during the protocol. In mentioned study, no significant differences in RPE between CAF and PLA conditions were observed. Similarly, Fogaca et al. [26] reported no difference in post-workout RPE between CAF and PLA conditions, but using the CR10 Borg Scale. In turn, Ziyaiyan et al. [28] recorded RPE on a scale of 1–10 and showed that the combination of CAF and NaHCO_3_ significantly reduced RPE in comparison to the control and PLA conditions. Similarly, other investigations, outside CrossFit/HIFT area, did not report any effect of CAF on RPE [26,50]. It can be concluded that the effects of CAF on RPE provide mixed results [48], when different exercise protocols are utilized. Crawford et al. [51] in a study on the RPE utilization in CrossFit suggested that a 15-point scale may be more appropriate in CrossFit athletes. Thus, since RPE is usually taken after the exercise bout, new studies might consider additional measurements of during-workout exertion.

Due to CNS activation, mainly through catecholamine release and adrenal cortex stimulation through corticoid release, CAF ingestion can influence HR at rest and during exercise. These processes result in adjustments to the cardiac autonomic modulation that induce consequent tachycardias [52]. With regard to meta-analysis by Benjamin et al. [53] it was concluded that HR increases during exercise and throughout the recovery after CAF ingestion. Moreover, it was indicated that CAF has the capacity to influence HR before, during, and after exercise. In our study, we measured total HR_mean_ during exercise and HR_max_, but surprisingly there were no differences between CAF doses and PLA. Unfortunately, only one other CAF CrossFit study measured HR changes during exercise protocol. Ziyaiyan et al. [28] found that HR_max_ at the end of the workout was significantly different between conditions, with emphasis on significantly greater value in CAF and CAF+NaHCO_3_ group compared to control. Based on the sports literature, CAF has equivocal effects on HR during periods of exercise. Our previous study in judo [18] showed that 6 and 9 mg_CAF_/kg_BM_ increased HR right after and 1 min after exercise test compared to 3 mg_CAF_/kg_BM_, and 9 mg_CAF_/kg_BM_ compared to PLA. Smirmaul et al. [54] reported the HR-increasing effect of CAF during cycling, suggesting that differences in HR_max_ were rather related to CAF than to exercise performance. Moreover, Bunsawat et al. [52] and Lopes-Silva et al. [55] showed higher HR in CAF group (HR_max_ and HR_mean_, respectively). On the contrary, Glaister et al. [40] found that during early stages of performance test CAF demonstrated lower HR. Other studies [56,57], similarly to our results, found no differences in HR_max_ between CAF and PLA groups.

Recent meta-analysis [8] on CAF and cognitive functions in sports revealed an improvement in response to CAF intake across a variety of cognitive domains. Analyzed studies showed a positive effect of CAF on relatively higher-order processes (visual selective attention measured by Stroop test and flanker task), with more pronounced effects in modalities with lower attention demands [8]. Research examined simple psychomotor speed (simple R_Time_), complex decision speed and decision-making time (choice R_Time_), complex response preparation speed (S-R incompatible CR_Time_) by simple visual R_Time_ test, motor CR_Time_ test and CR_Time_ test. It was concluded that CAF intake may improve simple R_Time_ and choice R_Time_, but it may depend on the exercise protocol. Importantly, it was revealed that a single acute dose of CAF is safe in some aspects of performance cognitive functions [8]. It was also suggested that CAF effect on R_Time_ may be a result of its effect on perceptual-attentional processes, rather than motor processes [58]. In our study, there were no significant differences in R_Time_ and M_Time_ between different CAF doses and PLA, although *MEDIUM* dose showed clinically important shorter pre-exercise R_Time_ and M_Time_. Interestingly, variability R_Time_ at this dose was maintained despite exercise, whereas the other doses and PLA or BASE resulted in a significant decrease in variability M_Time_ immediately after exercise. In study by Share et al. [59] no effect of CAF intake (2 and 4 mg_CAF_/kg_BM_) on clay target shooting, such as R_Time_ and target tracking time was detected. Church et al. [60] observed no difference in the ability to track multiple objects in response to 3 mg_CAF_/kg_BM_ 60 min before exercise. Although CAF may improve overall processing speed in tasks requiring higher-order functions, this cannot be attributed to specific effects on selective visual attention. Moreover, even though CAF may reduce response times and error rates in simple R_Time_ and CR_Time_, recent meta-analysis revealed that this may not translate into exercise [8]. Crowe et al. [61] using the simple visual R_Time_ test showed that CAF did not speed up cognitive R_Time_, although the effect might have been blunted because of the timing of CAF intake (90 min before the test). Russel et al. [62] analyzed CAF in chewing gum in rugby players and found no *treatment x time* interaction in simple R_Time_. Church et al. [60] and Share et al. [59] also found no differences between CAF and PLA in non-standardized measure to test separately upper and lower body R_Time_. Moreover, Ali et al. [63] found no effect of 6 mg_CAF_/kg_BM_ on complex decision-making capacity measured by CR_Time_ test. On the other hand, Bello et al. [10] found that 3.69 mg/kg_BM_ of CAF in soccer players improved simple psychomotor speed performance, CR_Time_ and cognitive load R_Time_. Hogervorst et al. [9] found that complex psychomotor speed and S-R incompatible choice speed, measured by motor CR_Time_ test, were significantly faster in *LOW* dose CAF than in PLA group. Interestingly, when assessing *CYP1A2* genotype variations no differences between AA homozygotes and C-allele carriers with regard to R_Time_ after CAF were found [64].

Our results revealed no effect of CAF or any differences between doses with regard to indices of P_Stab_ (Area95 and Vcop). Nevertheless, while there were no significant differences between doses and PLA, it is noteworthy (looking only at post-exercise results) that PLA was exceptionally better than any other dose, both for Vcop and for Area95, which could confirm the effect of CAF on the CNS. Unfortunately, there are no other studies on the effect of CAF on balance with regard to exercise performance tests. Results of the systematic review by Briggs et al. [11] on the effects of CAF ingestion on human standing balance indicated that younger participants’ balance was generally unaffected by CAF ingestion. Nevertheless, a significant impairment was observed in older participants. This suggests the possibility of an age-dependent effect of CAF on standing. Moreover, CAF should be considered as a potential confounding factor when assessing standing balance. Taking into account studies only in younger adults population, 100–500 mg of CAF 30–80 min after intake had no effect on balance when standing with eyes open or closed on a firm surface [65–68] or with a visual surround that moved with the body or during conditions designed to reduce proprioceptive information regarding body sway [67]. Waer et al. [68] reported no significant effect of 100 or 400 mg of CAF on standing with eyes open on a foam surface, but significantly reduced by 16% body sway derived from center of pressure with eyes closed following 100 mg of CAF.

### 4.1. Limitations

It should be noted that our research had some limitations. Firstly, we assessed the effect of the dose-dependent CAF supplementation in moderately trained athletes. It is possible that CAF effect may differ according to training experience and that highly trained/elite contestants may be more responsive to CAF, which was showed in our previous study in judo [18]. Still, the evidence is conflicting. Nevertheless, it is worth noting that since CrossFit/HIFT has become popular worldwide, most of the participants train moderately, thus in our opinion it is important to find new ergogenic solutions also for them. Secondly, we observed FGB practice effect between study meetings, which was previously seen by Stein et al. [27] in CrossFit participants. Interestingly, some authors [69] recommend at least two familiarization sessions to decrease the influence of this effect, especially in participants that are unaccustomed to exercise protocols. Nevertheless, on the contrary to other researchers, our studied participants were familiar with the efforts performed, which should have eliminated the learning effect. We also performed one familiarization session, which allowed the participants to get acquainted with the Department’s performance lab. Despite, the practice effect, CAF-induced improvement in specific performance may affect the result during competition. Moreover, we were not able to perform sex-dependent analyses differentiating CAF effects between sexes (uneven groups). This was caused by unwillingness of females to perform such a performance-demanding protocol. Finally, we accounted for practice effect only for FGB results. Nevertheless, each participant visited the laboratory five times and at each study visit we administered a single CAF or PLA dose to each participant. Moreover, it is possible that although we calculated the minimum sample size, a larger sample size would make certain differences more apparent.

### 4.2. Strengths

The unquestionable strength of our study was a multi-crossover design protocol and implementation of three different CAF doses, as well as PLA, in CrossFit/HIFT-trained practitioners with a complex assessment of specific performance, by a validated exercise test, and R_Time_ and P_Stab_, both before and after exercise. Secondly, we have taken into account SNPs of *CYP1A2* and *ADORA2A* genotypes and their role in CAF ergogenic effects (although the small number of participants per genotype may be a limitation). Thirdly, we are convinced that the implemented 7-day washout period was sufficient to remove CAF-induced ergogenic effect, since the half-life of CAF is 2.5–10 h [2]. Moreover, because the study protocols were performed strictly at the same time for each of the participants, we limited the effect of circadian rhythm on CAF activity and guaranteed the proper standardization. In addition, we calculated the minimum sample size, which was met by the number of participants who took part in the study. Furthermore, all study participants were habitual CAF users, who refrained from CAF-containing products before study visits, which limited the possibility of other factors influencing the results. We also ensured a full compliance with the intake of supplements and a thorough procedures’ standardization.

## 5. Conclusion

In conclusion, the present study does not confirm dose-dependent effect of CAF supplementation in moderately trained CrossFit/HIFT participants. There was no evidence of a difference between CAF doses and PLA on specific-performance, R_Time_, P_Stab_, RPE or HR, as well as *CYP1A2* and *ADORA2A* genotypes with regard to CAF supplementation. Nevertheless, the *MEDIUM* dose of CAF − 6 mg_CAF_/kg_BM_ induced clinically noticeable improvements in FGB, R_Time_ and M_Time_, which implicates that from the practical and athletic point of view this dose may be the most effective for CrossFit/HIFT practitioners.

## Data Availability

The results of the study are presented clearly, honestly, and without fabrication, falsification, or inappropriate data manipulation. The deidentified data are available from the corresponding author upon reasonable request.

## Clinical trial registration

This trial was registered prospectively at ClinicalTrials.gov (NCT03822663).
